# Volumetric MRI-based response assessment and prognostic value in newly diagnosed glioblastoma: RANO 2.0 versus mRANO versus RANO

**DOI:** 10.1093/noajnl/vdag032

**Published:** 2026-02-12

**Authors:** Johanna Heugenhauser, Wolfgang Orth, Manuel Sarcletti, Sarah Iglseder, Johannes Kerschbaumer, Christian F Freyschlag, Meinhard Nevinny-Stickel, Astrid Grams, Martha Nowosielski

**Affiliations:** Department of Neurology, Medical University Innsbruck, Innsbruck, Austria; Department of Neurology, Medical University Innsbruck, Innsbruck, Austria; Department of Radiation Oncology, Medical University Innsbruck, Innsbruck, Austria; Department of Neurology, Medical University Innsbruck, Innsbruck, Austria; Department of Neurosurgery, Medical University Innsbruck, Innsbruck, Austria; Department of Neurosurgery, Medical University Innsbruck, Innsbruck, Austria; Department of Radiation Oncology, Medical University Innsbruck, Innsbruck, Austria; Department of Neuroradiology & Neuroimaging Research Core Facility, Medical University Innsbruck, Innsbruck, Austria; Department of Neurology, Medical University Innsbruck, Innsbruck, Austria

**Keywords:** prognostic markers, radiologic response criteria, RANO 2.0, volumetric measurements

## Abstract

**Background:**

This retrospective study used volumetric MRI assessment to compare 3 response assessment criteria, namely, RANO (response assessment in neuro-oncology), mRANO (modified response assessment in neuro-oncology), and RANO 2.0, in patients with newly diagnosed glioblastoma (GB) treated with standard-of-care therapy. We evaluated whether progression-free survival (PFS), defined by each criterion, serves as a prognostic marker for overall survival (OS), and evaluated the impact of progression on patient outcome using landmark analyses.

**Methods:**

A total of 137 GB patients were included. Tumor volumes were assessed using semiautomatic 3D segmentation of contrast-enhancing and T2/fluid-attenuated inversion recovery lesions on serial MRI. The PFS was determined using RANO, mRANO, and RANO 2.0 criteria. Correlation between PFS and OS was evaluated using Spearman rank test. Differences in PFS and postprogression survival were tested with the Kruskal-Wallis test and corrected for multiple testing. Landmark analyses at 8 and 12 months were conducted to assess the prognostic effect of progression, with hazard ratios (HRs) derived from Cox models.

**Results:**

Median PFS differed significantly across criteria: 7.9 months (RANO), 11.3 months (mRANO), and 9.7 months (RANO 2.0). The correlation of PFS with OS was best with mRANO (ρ = 0.70), followed by RANO 2.0 (ρ = 0.66) and RANO (ρ = 0.50). The highest HR for death in patients with progressive disease was seen with RANO 2.0 (HR = 3.6), followed by mRANO (HR = 3.3), and RANO (HR = 3.3) (8-month landmark).

**Conclusion:**

mRANO and RANO 2.0 provided strong prognostic value in newly diagnosed GB patients. mRANO and RANO 2.0 showed strong correlation between PFS and OS, while RANO 2.0 demonstrated the strongest stratification of survival risk based on progression status at landmarks.

Key Points

- RANO 2.0 and mRANO show strong correlation between PFS and OS.
- RANO 2.0 provides best survival stratification at 8-month and 12-month landmarks.
- Median PFS varied significantly between the different response criteria.

Importance of the Study

In order to compare the responsiveness between different therapies among clinical trials and to differentiate between therapy-induced changes and true tumor progression, reliable response parameters are crucial. In this retrospective study, we compared 3 response assessment criteria: RANO (response assessment in neuro-oncology), mRANO (modified response assessment in neuro-oncology), and RANO 2.0 in patients with newly diagnosed glioblastoma treated with standard-of-care therapy. This work systematically evaluates the prognostic value of each assessment criteria using volumetric analysis and landmark survival models. We show that both mRANO and RANO 2.0 outperform RANO in correlating progression-free survival with overall survival. Notably, RANO 2.0 demonstrated the strongest ability to stratify survival risk based on progression status at clinically meaningful time points. These results suggest that RANO 2.0 may offer superior prognostic value for risk stratification and support its broader integration into clinical trials and routine practice to improve treatment response assessment and guide patient management.

Glioblastoma (GB), the most prevalent malignant primary brain tumor, is associated with a dismal median overall survival (OS) of approximately 15 months despite aggressive multimodal therapy.^1–4^ The current standard of care (SOC) for newly diagnosed GB includes maximal safe surgical resection followed by external beam radiation therapy with concomitant and adjuvant temozolomide, particularly in patients with O6-methylguanine-DNA methyltransferase (MGMT) promoter methylation.^1^^,^^5^^,^^6^ During adjuvant temozolomide, the addition of tumor-treating-fields (TTF), a modality delivering low-intensity, intermediate-frequency alternating electric fields can be considered.^2^

Although OS remains the gold standard endpoint in neuro-oncology trials, its use can be limited by long follow-up periods and potential confounding from postprogression treatments. Consequently, progression-free survival (PFS) has emerged as a frequently used surrogate endpoint.^7–9^ However, assessing PFS in GB poses several challenges. Treatment-related effects such as pseudoprogression (PsP), a transient increase in contrast enhancement on MRI resulting from therapy-induced vascular permeability changes, can mimic true tumor progression, especially within the first 3 months postradiotherapy.^10–12^ Other confounding factors include limitations in the assessment of nonenhancing tumor assessment,^13^^,^^14^ and variability in selecting the most appropriate baseline timepoint.^8^^,^^12^^,^^13^

In response to these challenges, various response assessment criteria have been developed. Established by a multidisciplinary working group of neuro-oncology experts, the RANO criteria aim to improve response assessments for high-grade glioma and to enhance the interpretation of clinical trials.^13^ Despite its advancements, the RANO criteria demonstrated limitations over time, including difficulties in interpreting postoperative changes, underrecognition of PsP, and the difficulty of evaluating nonenhancing tumor progression. To address these shortcomings, the modified RANO (mRANO) criteria were proposed, introducing important refinements such as mandatory confirmation of progression on follow-up imaging.^8^ Most recently, the RANO 2.0 criteria were developed based on real-world clinical data, rather than solely expert consensus.^12^^,^^15^ Key features of RANO 2.0 include using an MRI obtained 4 weeks after radiotherapy as baseline MRI in newly diagnosed GB and in the case of suspected PsP, a confirmatory MRI scan is only necessary if progression occurs within 12 weeks of radiation therapy.^12^

While RANO and mRANO have been applied across various clinical studies, variability in their application has led to inconsistencies in response assessments.^15–17^ Moreover, RANO 2.0 has not yet been widely implemented or validated in prospective trials. Therefore, the aim of this retrospective study is to directly compare RANO, mRANO, and RANO 2.0 criteria in a cohort of newly diagnosed GB patients, to determine which framework most reliably captures disease progression and provides clinically meaningful endpoints.

## Methods

### Study Population

**Figure 1.**
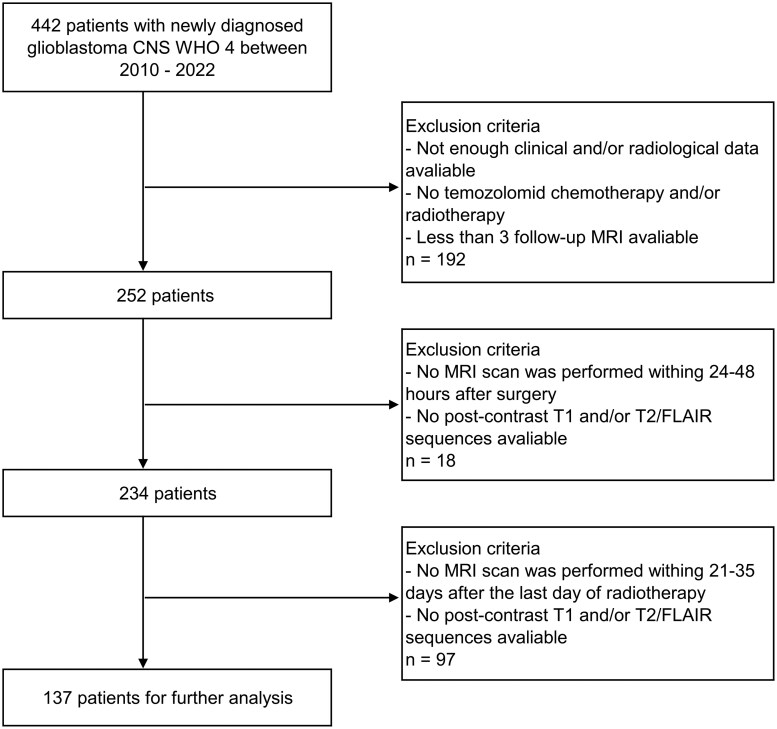
Flow chart of patient selection. Abbreviation: FLAIR, flair-attenuated inversion recovery.

In this retrospective analysis, imaging data from patients with a newly diagnosed GB isocitrate dehydrogenase (IDH) wildtype were evaluated. The IDH mutation was assessed by immunohistochemical staining in every patient. All included patients were treated with SOC at the Medical University of Innsbruck between 2010-2022. The SOC treatment included surgical resection followed by radiochemotherapy according to the “Stupp protocol” ± TTF.^1^^,^^2^  Figure 1 outlines the stepwise patient selection process for this retrospective cohort.

The study was reviewed and approved by the local independent ethics committee and institutional review board (EK Nr: 1364/2023).

### Magnetic Resonance Imaging

Multiple MRI systems were used, all operating at a 1.5T or 3T field strength. A 3D T1-weighted imaging protocol including magnetization-prepared rapid acquisition with gradient echo (MP-RAGE) MRI sequences or T1-weighted spin-echo (SE) images with contrast enhancement (0.1 mmol/kg gadopentetate dimeglumine) was performed, resulting in images of similar resolution, with a slice thickness of at least 1.5 mm. All patients underwent axial T2-weighted or fluid-attenuated inversion recovery (FLAIR)-weighted imaging with a slice thickness of at least 2 mm. Because RANO and mRANO/RANO 2.0 criteria define different time points for baseline MRI, each included patient was required to have both of the following imaging studies: (1) a postoperative baseline MRI for RANO criteria, performed within 24 to 48 hours after surgery; and (2) a baseline MRI for mRANO and RANO 2.0 criteria, conducted 21 to 35 days after the completion of radiotherapy. Follow-up MRIs were performed every 3 months, with each patient undergoing at least 3 follow-up scans after the postoperative MRI. In this post hoc analysis, imaging data were analyzed until death/loss to follow-up of each patient.

### Radiologic Response Assessment

Radiological response assessment was independently performed by 2 readers (J.H. and W.O.), who were blinded to patients’ clinical data and outcomes. In cases of disagreement during consensus review, a third expert neuroradiologist (A.G.) was consulted. Although current guidelines are still based on 2-dimensional assessment,^8^^,^^12^^,^^13^ the literature increasingly debates the potential superiority of 3-dimensional (3D) methods.^18–20^ In this analysis, we therefore applied a purely 3D volumetric approach. As the RANO criteria do not specify volumetric measurement guidelines, we adopted the framework described by Kickingereder et al^21^ and modified RANO for volumetric criteria. While their study relied on a fully automated assessment, we used a semiautomatic approach but applied the same volumetric thresholds (eg, a 100% increase in the total volume of nonenhancing lesions to define progression), which are provided in Table S1.

The postoperative MRI (performed 24-48 hours after surgery) served as the baseline scan for RANO criteria,^13^ while the MRI acquired approximately 4 weeks after the end of radiotherapy was used as baseline MRI for mRANO and RANO 2.0 criteria.^8^^,^^12^ The contrast-enhancing tumor volume (excluding dura, blood vessels, the resection ­cavity and/or central necrotic area) was calculated on postgadolinium isovoxel T1-weighted MRI sequences. T2/FLAIR enhancing abnormalities (excluding the contrast-enhancing area as well as the necrotic area of the tumor mass) were calculated on T2-/FLAIR-weighted images. In case of more than one lesion (maximum of 5 target lesions), each volume was summed up to calculate one total tumor volume. Measurable disease was defined as a tumor volume ≥1 cm^3^.^8^^,^^12^ Tumor segmentation was performed using a semiautomated active contour method (ITK-SNAP 3.8.0), which demonstrated excellent reliability and high efficiency of 3D segmentation.^17^^, ^^22^

**Figure 2.**
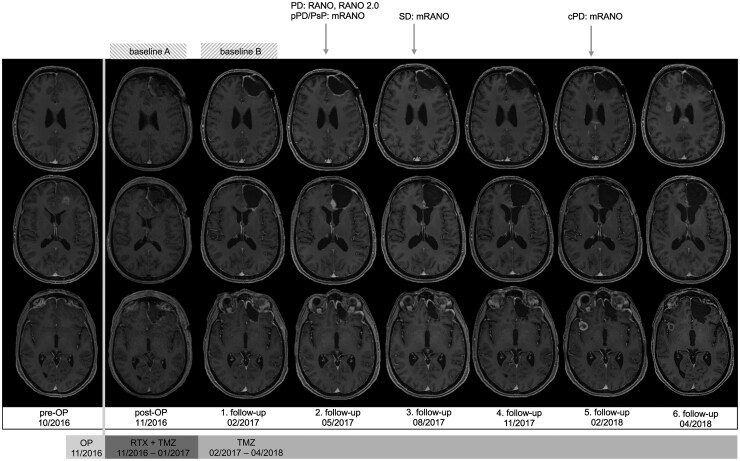
Response assessment on a representative patient. Patient (GB-0110): post-op MRI serves as baseline A for RANO, while the postradiation MRI/1.follow-up (21-35 days after the last day of radiotherapy) serves as baseline B for mRANO and RANO 2.0. At the second follow-up MRI, tumor progression is suspected because a new measurable lesion appeared (<1 cm^3^), and PD is defined according to RANO. Since this scan was obtained more than 12 weeks after completion of radiotherapy, PD is also defined according to RANO 2.0. In contrast, mRANO classifies this finding as preliminary PD. At the third follow-up MRI, a reduction in tumor volume is observed; therefore, SD is defined according to mRANO, and the prior MRI (second follow-up) is retrospectively classified as pseudoprogression. Tumor volume remains stable through the fourth follow-up. However, at the fifth follow-up MRI, a new lesion is detected, leading to a diagnosis of PD according to mRANO. Abbreviations: cPD, confirmed progressive disease; mRANO, modified response assessment in neuro-oncology; PD, progressive disease, pPD, preliminary progressive disease, PsP, pseudoprogression; RANO, response assessment in neuro-oncology; RTX, radiotherapy; SD, stable disease; TMZ, temozolomide.

Each patient was classified as complete response (CR), partial response (PR), stable disease (SD), or progressive disease (PD) according to the 3 different assessment criteria at every available follow-up MRI scan. As defined by mRANO and RANO 2.0, PsP was diagnosed when the follow-up MRI (performed 4-8 weeks after suspected PD) did not demonstrate an additional ≥40% increase in tumor volume.^13^ Since our focus was on validating imaging parameters for response assessment criteria, we did not evaluate neurological function or corticosteroid dosage, despite these being recommended by the response assessment criteria. The date of the first follow-up MRI indicating progressive disease (PD), or confirmed PD in the case of mRANO and RANO 2.0, was defined as the date of progression. While RANO does not mandate a confirmatory MRI for suspected PD, it strongly advises against defining PD within 12 weeks of radiotherapy. Accordingly, in our study, any suspicion of PD within this interval required mandatory assessment of subsequent MRIs. The PD was dated to the MRI at which potential progression was first identified only when follow-up imaging demonstrated further tumor growth. Patients who showed stable disease (SD) at their final available scan were censored at the date of that scan. If a re-resection was performed, the last MRI prior to surgery was considered the date of progression, if progression had not been identified earlier, as all re-resected cases showed histopathological confirmed tumor progression. As bevacizumab can reduce contrast enhancement on postcontrast MRI,^14^^,^^23^ the last MRI scan prior to the initiation of second-line therapy with bevacizumab was used as progression date, if progressive disease had not already been diagnosed previously according to any of the assessment criteria. The PFS was calculated from the date of the preoperative MRI (i.e. date of diagnosis, 1-14 days prior to surgery) to the date of progression according to the different assessment criteria. Postprogression survival (PPS) was calculated from the date of progression according to the different assessment criteria to the date of death or last follow-up. We calculated PPS to evaluate which assessment criteria is superior in predicting OS. We assumed, if PPS is short, the time point of progression assessed by MRI is a reliable surrogate endpoint. The OS was calculated from the date of the preoperative MRI to the date of death or last follow-up. Nineteen patients who were lost to follow-up or were alive at the end of the study were censored at the date of their last documented visit. A summary of the different assessment criteria is displayed in Table S1. Figure 2 provides a visual example of how response status was determined in a single representative patient.

### Statistical Analysis

Results of OS, PFS, and PPS are reported as median with interquartile range (IQR). Distribution of OS, PFS, and PPS were calculated by the Shapiro-Wilk test. Differences in PFS and PPS between criteria were assessed using the Kruskal-Wallis test, followed by Dunn test with multiple testing correction (Bonferroni adjustment). For correlation analysis between PFS and OS, the Spearman rank test was used. Landmark analysis was carried out to detect whether progression at the 8-month or 12-month landmark has an influence on survival. Patients who had died prior to these specific landmarks were excluded from analysis. At the 8-month and 12-month landmark, response (PD or SD) according to each assessment criteria was assessed for each patient and residual survival time was calculated (date of MRI at 8-month or 12-month after presurgery MRI to date of death/loss to follow-up). Differences in median OS between PD and SD according to each assessment criteria at specific landmark were calculated by Mann-Whitney U test. Cox proportional hazards models were fitted using progression status (PD vs SD) at fixed 8-month and 12-month landmarks as the main covariate. Survival time was defined as time from the landmark onward, and only patients alive and uncensored at the landmark were included in each respective model. Hazard ratios (HRs) and 95% confidence intervals (CIs) were reported. Statistical analyses were ­performed using R software version 4.5.0.

## Results

### Patient Characteristics

In total, 442 patients were diagnosed with a GB CNS WHO Grade 4 at Medical University Innsbruck between 2010 and 2022. The required inclusion criteria for this retrospective analysis were met for 137 (56 women, 81 men) patients. All patients had histologically confirmed GB according to the latest 2021 WHO classification of adult brain tumors.^24^ Gross total resection (absence of all residual enhancing tumor) was achieved in 120 patients (87.6%), while 17 patients (12.4%) underwent partial resection. All patients received external beam radiation therapy (60 Gy) with concomitant temozolomide, followed by a median of 6 cycles of adjuvant temozolomide. Median time between surgery and the start of concomitant radiochemotherapy was 38 days (IQR: 12.0). The TTF was administered in 23 patients (16.8%). In Table 1, clinical and demographic information of the patient cohort is given.

**Table 1.** Clinical and demographic information of the study cohort

Sex, n (%)	Male	81 (59.1)
	Female	56 (40.9)
Median age at diagnosis, y (IQR)		58.88 (14.54)
Median overall survival, mo (IQR)		20.74 (18.32)
Survival end of study, n (%)	Death	118 (86.1)
	Alive	14 (10.2)
	Unknown	5 (3.7)
KPS at baseline, n (%)	≥70	125 (91.2)
	<70	12 (8.8)
MGMT promoter, n (%)	Methylated	46 (33.6)
	Unmethylated	53 (38.7)
	Unknown	38 (27.7)
IDH 1 mutation, n (%)	Yes	0 (00.0)
	No	137 (100.0)
Side of tumor, n (%)	Right	68 (49.6)
	Left	21 (15.3)
	Bilateral/central	48 (35.1)
Tumor location, n (%)	Frontal	40 (29.2)
	Temporal	43 (31.4)
	Parietal	36 (26.3)
	Occipital	18 (13.1)

Abbreviations: IDH 1, isocitrate dehydrogenase 1; IQR, interquartile range; KPS, Karnofsky performance status; MGMT, O-6-methylguanine-DNA methyltransferase; n, number of patients.

### PFS and PPS

Median PFS was significantly longer with mRANO (11.3 months; IQR: 11.8) and RANO 2.0 (9.7 months; IQR: 10.4) compared with RANO (*P* *<* .001), while PFS was shortest with RANO (7.9 months; IQR: 10.0). No significant difference in PFS was observed between mRANO and RANO 2.0 (*P* = .890).

The PPS was significant shorter with mRANO (8.2 months; IQR: 10.4) and RANO 2.0 (9.2 months; IQR: 10.4) compared with PPS with RANO (*P* = .029 and *P* *<* .001), while PPS was longest with RANO (13.4 months; IQR: 14.8). No significant difference in PPS was observed between mRANO and RANO 2.0 (*P* = .702).

**Figure 3.**
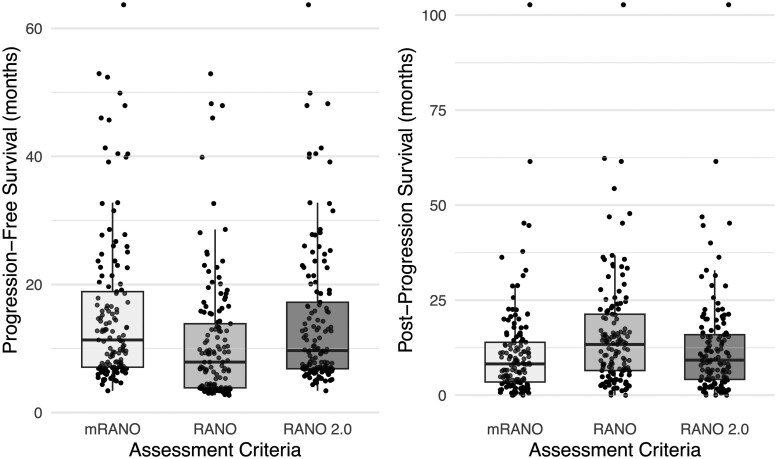
Progression-free survival and postprogression survival of different assessment criteria.

Figure 3 illustrates the distribution of PFS and PPS values by the different assessment methods.

According to the RANO criteria, progression can be defined by an increase in nonenhancing lesions alone. Although no exact threshold is specified, we applied a >100% increase in volume to define PD. In our cohort, with RANO only 8 patients (5.8%) showed progression of nonenhancing lesions prior to the appearance of increased contrast-enhancing lesions.

### Correlation Between PFS and OS

The strongest correlation between OS and PFS was observed with mRANO (ρ = 0.70, *P* < .001), followed by RANO 2.0 (ρ = 0.66, *P* < .001). In contrast, RANO showed only a weak correlation between OS and PFS (ρ = 0.50, *P* < .001). Scatter plots illustrating the correlation between PFS and OS for each assessment criterion are provided in Figure S1.

### Prognostic Impact of Stable Versus Progressive Disease at Landmarks

In total, at the 8-month landmark and at the 12-month landmark, 137 patients (100%) and 121 (88.3%) patients were analyzed, respectively. At both landmarks, median OS between SD and PD was significantly different for each assessment criteria. Table 2 presents the median OS at both landmark time points, stratified by assessment method. It also includes the proportion of patients classified as having SD or PD according to each assessment criterion. We added Wilcoxon rank‑sum *P* values comparing OS between progressive versus stable patients for each criterion. Given the wide IQRs, a nonparametric test was used.

**Table 2.** Landmark analysis

Response criteria	SD			PD			
	n (%)	Median OS (mo)	IQR	n (%)	Median OS (mo)	IQR	*P*
8-mo landmark							
RANO	66/137 (48.2)	32.60	22.00	71/137 (51.8)	18.40	9.76	<.001
mRANO	95/137 (69.3)	27.20	18.60	42/137 (30.7)	16.20	7.50	<.001
RANO 2.0	84/137 (61.3)	29.20	22.00	53/137 (38.7)	17.70	8.68	<.001
12-mo landmark							
RANO	48/121 (39.7)	32.7	22.20	73/121 (60.3)	18.80	10.40	<.001
mRANO	73/121 (60.3)	32.5	20.50	58/121 (47.9)	16.90	7.95	<.001
RANO 2.0	63/121 (52.1)	33.2	20.3	58/121 (47.9)	18.60	7.89	<.001

Abbreviations: IQR, interquartile range; mRANO, modified response assessment in neuro-oncology; n, number of patients; PD, progressive disease; RANO, response assessment in neuro-oncology; SD, stable disease.

**Figure 4.**
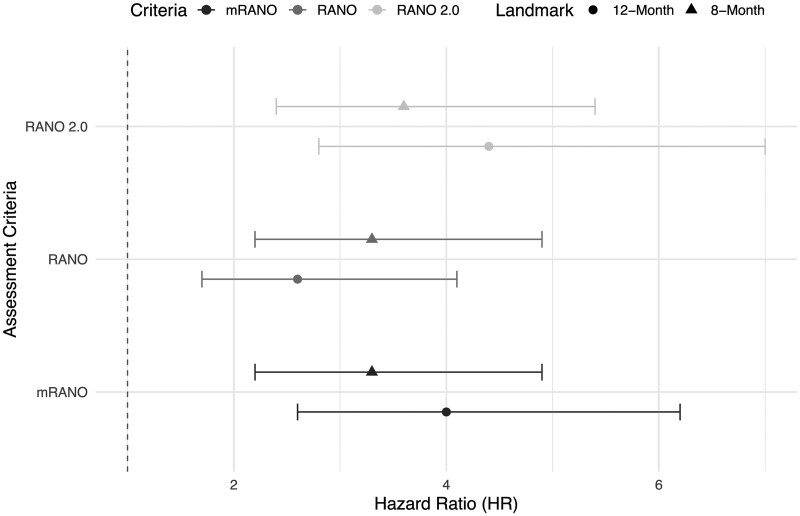
Forest plot of hazard ratios for progressive disease at landmarks.

At the 8-month landmark, patients classified as having progressive disease had a significantly increased risk of death according to all 3 criteria. The HRs were 3.3 (95% CI: 2.2-4.9; *P* < .001) for RANO, 3.3 (95% CI: 2.2-4.9; *P* < .001) for mRANO, and 3.6 (95% CI: 2.4-5.4; *P* < .001) for RANO 2.0. At the 12-month landmark, HRs increased for mRANO (4.0, 95% CI: 2.6-6.2; *P* < .001) and RANO 2.0 (4.4, 95% CI: 2.8-7.0; *P* < .001), but decreased for RANO (2.6, 95% CI: 1.7-4.1; *P* < .001). These HRs are summarized visually in Figure 4. Corresponding Kaplan-Meier survival curves for SD versus PD at each landmark, stratified by assessment criteria, are presented in Figure S2.

### Pseudoprogression

Based on imaging, overall, 24 patients (17.5%) were classified as having PsP, evaluated by mRANO and RANO 2.0. The median time of developing PsP after radiotherapy was 19.1 weeks (IQR: 5.7), while only 4 patients (2.9%) exhibited PsP in the first 12 weeks postradiotherapy.

The median OS for patients with PsP was 23.3 months (IQR: 21.8), compared with 19.6 months (IQR: 18.0) in patients without PsP. This difference was not statistically significant (*P* = .120).

## Discussion

Accurate stratification of disease status in GB requires both clinical and imaging evaluation. MRI remains the gold standard, and the RANO criteria provide a standardized framework for response assessment in trials and practice. These criteria have been refined over the past decade to address challenges such as PsP, as distinguishing true progression from treatment effects is critical to avoid premature therapy changes. Recently, a revised version of the RANO criteria, termed RANO 2.0, was proposed. Although RANO 2.0 has been increasingly adopted in both academic centers and clinical trials, the appearance of clinical trial reports using these updated criteria will require additional time.^12^ In this retrospective study, we compare 3 response assessment criteria in neuro-oncology (RANO, mRANO, and RANO 2.0,^8^^,^^12^^,^^13^) to evaluate their performance in accurately assessing treatment response in newly diagnosed GB patients treated with the current SOC.^1^

When evaluating PFS as the endpoint, both mRANO and RANO 2.0 demonstrated significantly longer PFS compared with RANO. Furthermore, the correlation between PFS and OS was stronger in mRANO and RANO 2.0, compared with RANO. Although our findings are only partly in line with previous studies comparing response assessment criteria, important differences in study populations should be noted.^16^^,^^17^ The large study by Youssef et al,^15^ which included 526 newly diagnosed GB patients and partly informed the development of RANO 2.0, investigated a patient population most comparable with ours. In that study, similar correlations between PFS and OS were observed when applying RANO and mRANO. In ­contrast, in a cohort of 41 patients with recurrent GB treated with an experimental immunotoxin, PFS was significantly shorter using RANO compared with mRANO. Moreover, the correlation between OS and PFS was strong using mRANO but weak using RANO.^16^ In another study of newly diagnosed patients treated with SOC with or without tumor lysate-charged dendritic cells, mRANO also showed a stronger correlation between OS and PFS as well as a longer PFS when compared with MacDonald, RANO, and iRANO.^17^ Thus, while our findings align with prior studies in showing longer PFS under mRANO compared with RANO and a stronger correlation between PFS and OS with mRANO, they differ from Youssef et al in the relative strength of the correlation between PFS and OS. However, the latter 2 study cohorts included patients treated with immunotherapy, which may be associated with higher rates of PsP. These differences should be kept in mind when interpreting consistency across studies. While PFS remains widely used in GB studies, it cannot be considered a robust or fully reliable endpoint due to substantial imaging-related limitations.^7–9^ These issues, already evident with RANO and still partly present in RANO 2.0, contribute to the weak correlation with OS and have led regulators to refrain from accepting PFS as a primary endpoint in newly diagnosed GB trials. One controversial topic is the ongoing debate regarding the optimal timepoint for establishing a baseline scan. While earlier response assessment criteria use a postoperative MRI (performed within 24-48 hours after surgery) as baseline,^13^^,^^25^^,^^26^ more recent response assessment criteria advocate for using an MRI acquired within 4 weeks after the completion of radiotherapy.^8^^,^^12^ The postradiotherapy baseline scan, as used in RANO and RANO 2.0, was introduced to minimize misclassification due to PsP and potential effects of variable corticosteroid dosing.^12^ This issue was explicitly addressed in a study by Youssef et al, who included 526 patients with newly diagnosed GB. The authors found that using the postradiotherapy scan resulted in a stronger correlation between PFS and OS, although the difference did not reach statistical significance.^15^ In line with their general observation, we also found that using postradiotherapy MRI as baseline resulted in a stronger correlation between PFS and OS, indicating that this timepoint provides a more reliable reference for assessing treatment response and disease progression.^15^

Another factor complicating response assessment and influencing PFS is therapy-related PsP after radiochemotherapy.^11–13^^,^^27^^,^^28^ Although this issue was initially acknowledged in the RANO criteria and a strong recommendation against considering PD during the first 12 weeks after radiotherapy is given, no specific protocol was established for managing suspected PsP.^13^ In contrast, the mRANO and RANO 2.0 criteria have introduced further steps to address this challenge by requiring confirmation scans when progression is first observed on MRI.^8^^,^^12^ Unlike mRANO, RANO 2.0 requires confirmation scans only within the first 12 weeks after radiotherapy, as the study by Youssef et al showed no added benefit in confirming progression beyond this early post-treatment period.^12^^,^^15^ The rate of PsP in our cohort is comparable with previous reports^29^; however, the median time to PsP was 19.1 weeks after radiotherapy, which is later than typically described.^15^ As a result, several patients were classified as having PD under the RANO 2.0 criteria because confirmation scans are not required beyond the 12-week post-radiotherapy window. This may partially account for the slightly weaker correlation between PFS and OS observed with RANO 2.0 compared with mRANO.

A likely explanation for the later observed onset of PsP in our study is the extended inclusion period (2010-2022), which introduced variability in follow-up imaging frequency, particularly in the earlier years. Although all patients underwent MRI 21 to 35 days after completing radiotherapy, subsequent scans were not consistently performed at strict 3-month intervals. As a result, PsP that developed earlier may not have been detected at the time of its true onset and was instead identified at a later scan. This pattern likely reflects delayed detection rather than a biological delay in the development of PsP. These findings underscore the somewhat artificial nature of the 12-week cutoff and suggest that rigid adherence to this threshold may lead to misclassification in clinical practice. A more flexible, biology-guided interpretation of early post-treatment imaging may therefore be warranted.

With landmark analysis, we stratified the risk of death according to each assessment criteria. Interestingly, at both the 8-month and 12-month landmarks, patients classified as having progressive disease according to the RANO 2.0 criteria demonstrated the highest risk of death, potentially indicating that RANO 2.0 may provide superior prognostic stratification and more effectively capture clinically meaningful progression.

There are some limitations to consider. First, precise imaging assessment may have been affected by the use of older MRI protocols. For example, only T1-weighted SE images were available for certain patients. A further limitation of our study is the definition of progression within the first 12 weeks after completion of radiotherapy.^13^ The original RANO recommendations do not provide a standardized workflow for how to consistently evaluate suspected cases in this setting. In our retrospective cohort, advanced imaging techniques and tissue confirmation were not uniformly available, and we therefore evaluated subsequent brain MRI to determine whether the findings represented true progression or PsP. Furthermore, in contrast to current response assessment criteria, we did not evaluate neurological function, patient-reported quality of life, or corticosteroid dosage, as these data were inconsistently documented. Hence, our retrospective study serves primarily as a validation of imaging parameters for response assessment in neuro-oncology. Prospective validation of the most recent RANO 2.0 criteria in larger, well-characterized cohorts including clinical parameters and information concerning corticosteroid use will be essential to support broader clinical implementation.

## Conclusion

In this retrospective cohort of newly diagnosed GB patients, both mRANO and RANO 2.0 demonstrated strong prognostic value, with correlations between PFS and OS superior to RANO. In our cohort, PsP tended to occur relatively late and, therefore, not all cases were captured by RANO 2.0. Nonetheless, at both the 8-month and 12-month landmarks, patients classified as progressive according to RANO 2.0 had the highest risk of death, suggesting that it stratifies survival risk effectively. However, as the prognostic performance of mRANO and RANO 2.0 was relatively similar in our analyses, we cannot conclude superiority of one over the other. Given that RANO 2.0 incorporates many elements of mRANO, comparable outcomes are expected.

## Supplementary Material

vdag032_Supplementary_Data

## Data Availability

The data will be made available upon reasonable request.

## References

[1] Stupp R , Mason WP, van den Bent MJ, et al Radiotherapy plus concomitant and adjuvant temozolomide for glioblastoma. N Engl J Med. 2005;352:987-996. 10.1056/NEJMoa043330 15758009

[2] Stupp R , Taillibert S, Kanner A, et al Effect of tumor-treating fields plus maintenance temozolomide vs maintenance temozolomide alone on survival in patients with glioblastoma: a randomized clinical trial. Jama. 2017;318:2306-2316. 10.1001/jama.2017.18718 29260225 PMC5820703

[3] Herrlinger U , Tzaridis T, Mack F, et al Lomustine-temozolomide combination therapy versus standard temozolomide therapy in patients with newly diagnosed glioblastoma with methylated MGMT promoter (CeTeG/NOA-09): a randomised, open-label, phase 3 trial. Lancet. 2019;393:678-688. 10.1016/S0140-6736(18)31791-4 30782343

[4] Ostrom QT , Patil N, Cioffi G, et al CBTRUS statistical report: primary brain and other central nervous system tumors diagnosed in the United States in 2013-2017. Neuro Oncol. 2020;22:iv1-iv96. 10.1093/neuonc/noaa200 33123732 PMC7596247

[5] Wen PY , Weller M, Lee EQ, et al Glioblastoma in adults: a Society for Neuro-Oncology (SNO) and European Society of Neuro-Oncology (EANO) consensus review on current management and future directions. Neuro Oncol. 2020;22:1073-1113. 10.1093/neuonc/noaa106 32328653 PMC7594557

[6] Weller M , van den Bent M, Preusser M, et al EANO guidelines on the diagnosis and treatment of diffuse gliomas of adulthood. Nat Rev Clin Oncol. 2021;18:170-186. 10.1038/s41571-020-00447-z 33293629 PMC7904519

[7] Lamborn KR , Yung WKA, Chang SM, et al Progression-free survival: an important end point in evaluating therapy for recurrent high-grade gliomas. Neuro Oncol. 2008;10:162-170. 10.1215/15228517-2007-062 18356283 PMC2613818

[8] Ellingson BM , Wen PY, Cloughesy TF. Modified criteria for radiographic response assessment in glioblastoma clinical trials. Neurotherapeutics. 2017;14:307-320. 10.1007/s13311-016-0507-6 28108885 PMC5398984

[9] Han K , Ren M, Wick W, et al Progression-free survival as a surrogate endpoint for overall survival in glioblastoma: a literature-based meta-analysis from 91 trials. Neuro Oncol. 2014;16:696-706. 10.1093/neuonc/not236 24335699 PMC3984546

[10] Brandsma D , Stalpers L, Taal W, Sminia P, van den Bent MJ. Clinical features, mechanisms, and management of pseudoprogression in malignant gliomas. Lancet Oncol. 2008;9:453-461. 10.1016/S1470-2045(08)70125-6 18452856

[11] Taal W , Brandsma D, de Bruin HG, et al Incidence of early pseudo-progression in a cohort of malignant glioma patients treated with chemoirradiation with temozolomide. Cancer. 2008;113:405-410. 10.1002/cncr.23562 18484594

[12] Wen PY , van den Bent M, Youssef G, et al RANO 2.0: update to the response assessment in neuro-oncology criteria for high- and Low-Grade gliomas in adults. J Clin Oncol. 2023;41:5187-5199. 10.1200/jco.23.01059 37774317 PMC10860967

[13] Wen PY , Macdonald DR, Reardon DA, et al Updated response assessment criteria for high-grade gliomas: response assessment in neuro-oncology working group. J Clin Oncol. 2010;28:1963-1972. 10.1200/JCO.2009.26.3541 20231676

[14] Nowosielski M , Wiestler B, Goebel G, et al Progression types after antiangiogenic therapy are related to outcome in recurrent glioblastoma. Neurology. 2014;82:1684-1692. 10.1212/WNL.0000000000000402 24727314

[15] Youssef G , Rahman R, Bay C, et al evaluation of standard response assessment in neuro-oncology, modified response assessment in neuro-oncology, and immunotherapy response assessment in neuro-oncology in newly diagnosed and recurrent glioblastoma. J Clin Oncol. 2023;41:3160-3171. 10.1200/JCO.22.01579 37027809

[16] Ellingson BM , Sampson J, Achrol AS, et al Modified RANO, immunotherapy RANO, and standard RANO response to convection-enhanced delivery of IL4R-targeted immunotoxin MDNA55 in recurrent glioblastoma. Clin Cancer Res. 2021;27:3916-3925. 10.1158/1078-0432.ccr-21-0446 33863808 PMC8282697

[17] Heugenhauser J , Galijasevic M, Mangesius S, et al MRI response assessment in glioblastoma patients treated with dendritic-cell-based immunotherapy. Cancers (Basel). 2022;14 10.3390/cancers14061579 PMC8946797 35326730

[18] Dempsey MF , Condon BR, Hadley DM. Measurement of “tumor” size in recurrent malignant glioma: 1D, 2D, or 3D? AJNR Am J Neuroradiol. 2005;26:770-776. 15814919][Mismatch 15814919 PMC7977136

[19] Gahrmann R , van den Bent M, van der Holt B, et al Comparison of 2D (RANO) and volumetric methods for assessment of recurrent glioblastoma treated with bevacizumab: a report from the BELOB trial. Neuro Oncol. 2017;19:853-861. 10.1093/neuonc/now311 28204639 PMC5464446

[20] Shah GD , Kesari S, Xu R, et al Comparison of linear and volumetric criteria in assessing tumor response in adult high-grade gliomas. Neuro Oncol. 2006;8:38-46. 10.1215/S1522851705000529 16443946 PMC1871928

[21] Kickingereder P , Isensee F, Tursunova I, et al Automated quantitative tumour response assessment of MRI in neuro-oncology with artificial neural networks: a multicentre, retrospective study. Lancet Oncol. 2019;20:728-740. 10.1016/S1470-2045(19)30098-1 30952559

[22] Yushkevich PA , Piven J, Hazlett HC, et al User-guided 3D active contour segmentation of anatomical structures: significantly improved efficiency and reliability. Neuroimage. 2006;31:1116-1128. 10.1016/j.neuroimage.2006.01.015 16545965

[23] Huang RY , Rahman R, Ballman KV, et al The impact of T2/FLAIR evaluation per RANO criteria on response assessment of recurrent glioblastoma patients treated with bevacizumab. Clin Cancer Res. 2016;22:575-581. 10.1158/1078-0432.CCR-14-3040 26490307

[24] Louis DN , Perry A, Wesseling P, et al The 2021 WHO classification of tumors of the central nervous system: a summary. Neuro Oncol. 2021;23:1231-1251. 10.1093/neuonc/noab106 34185076 PMC8328013

[25] Macdonald DR , Cascino TL, Schold SC Jr., Cairncross JG. Response criteria for phase II studies of supratentorial malignant glioma. J Clin Oncol. 1990;8:1277-1280. 10.1200/JCO.1990.8.7.1277 2358840

[26] Okada H , Weller M, Huang R, et al Immunotherapy response assessment in neuro-oncology: a report of the RANO working group. Lancet Oncol. 2015;16:e534-e542. 10.1016/S1470-2045(15)00088-1 26545842 PMC4638131

[27] Brandes AA , Franceschi E, Tosoni A, et al MGMT promoter methylation status can predict the incidence and outcome of pseudoprogression after concomitant radiochemotherapy in newly diagnosed glioblastoma patients. J Clin Oncol. 2008;26:2192-2197. 10.1200/JCO.2007.14.8163 18445844

[28] Hagiwara A , Oughourlian TC, Cho NS, et al Diffusion MRI is an early biomarker of overall survival benefit in IDH wild-type recurrent glioblastoma treated with immune checkpoint inhibitors. Neuro Oncol. 2022;24:1020-1028. 10.1093/neuonc/noab276 34865129 PMC9159421

[29] Galldiks N. Diagnosing pseudoprogression in glioblastoma: a challenging clinical issue. Neurooncol Pract. 2024;11:1-2. 10.1093/nop/npad078 38222056 PMC10785576

