# Supplementary material for: Volumetric MRI-based response assessment and prognostic value in newly diagnosed glioblastoma: RANO 2.0 versus mRANO versus RANO
Source: Neurooncol Adv. 2026 Feb 12;8(1):vdag032. doi: 10.1093/noajnl/vdag032 (PMC12986764; doi:10.1093/noajnl/vdag032)

Supplementary Table 1: Different Response Assessment Criteria

	Complete response (CR)	Partial response (PR)	Stable disease (SD)	Progressive disease (PD)	
RANO	• disappearance of enhancing measurable and non-measurable<br>• no new lesion<br>• stable/improved non-enhancing (T2/FLAIR)<br><br>• steroid: off<br>• clinical: improved/stable	• ≥65% decrease in total volume of measurable enhancing<br>• no progress of non-measurable<br>• stable/improved non-enhancing (F2/FLAIR)<br><br>• steroid: stable/reduced<br>• clinical: stable/improved	• stable non-enhancing (F2/FLAIR)<br>• stable enhancing<br>• no new lesion<br>• best response for patients with non-measurable disease at baseline<br><br>• steroid: stable/improved<br>• clinical: stable	• ≥40% increase in total volume of enhancing<br>• new measurable lesion<br>• 100% increase in total volume in non-enhancing (T2/FLAIR)*<br>• change from non-measurable to measurable lesion<br><br>• steroid: stable/increased<br>• clinical: deterioration	• baseline MRI: after surgery<br>• measurable disease: ≥1 cm^3<br>• increase in non-measurable: >5mm or ≥25% sum of products<br>• strong recommendation against considering PD in first 12 weeks of RT → follow-up MRI
mRANO	1.MRI: Preliminary CR<br>• disappearance of all measurable and non-measurable disease<br>• no new lesion<br><br>2.MRI (4-8 weeks later):<br>• if continuous disappearance: durable CR<br>• if measurable enhancing: preliminary PD/ pseudoresponse (1.MRI)<br><br>• steroid: off<br>• clinical: improved/stable	1.MRI Preliminary PR<br>• ≥65% decrease in total volume of measurable enhancing<br>• no new lesion<br><br>2.MRI (4-8 weeks later):<br>• if SD, PR or CR: durable PR<br>• if PD: preliminary PD/ pseudoresponse (1.MRI)<br><br>• steroid: stable/reduced<br>• clinical: stable/improved	• stable enhancing<br>• no new lesion<br>• best response for patients with non-measurable disease at baseline<br><br>• steroid: stable/improved<br>• clinical: stable	1.MRI: Preliminary PD<br>• new measurable lesion<br>• ≥40% increase in total volume of enhancing<br><br>2.MRI (4-8 weeks later):<br>• ≥40% increase in total volume: confirmed PD<br>• if SD or PR/CR: pseudoprogression (1.MRI)<br><br>• steroid: stable/increased<br>• clinical: deterioration	• baseline MRI: after radiotherapy<br>• no T2/FLAIR<br>• only confirmed PD stop therapy<br>• PD date is backdated to when first assumed
RANO 2.0	1.MRI: Preliminary CR<br>• disappearance of all measurable, non-measurable and nontarget disease<br>• no new lesion<br><br>2.MRI (4-8 weeks later):	1.MRI Preliminary PR<br>• ≥65% decrease in total volume of measurable enhancing<br>• no new lesion<br>• no increase in nontarget or non-measurable lesions<br><br>2.MRI (4-8 weeks later):<br>• if SD, PR or CR: durable PR	• stable enhancing<br>• no new lesion<br>• no increase in nontarget or non-measurable lesions<br>• best response for patients with non-measurable disease at baseline	1.MRI: Preliminary PD<br>• new measurable lesion<br>• ≥40% increase in total volume of enhancing<br>• definite leptomeningeal disease<br>• clear progression of non-measurable or nontarget<br>• failure to return to evaluation because of death or deteriorating condition	• baseline MRI: 4 weeks (21-35 days) from the end of radiotherapy<br>• if confirmation scan required, new measurable enhancing disease added to total volume. Only PD if confirmed by 2. MRI ≥4 weeks with additional ≥40% increase in volume<br>• if multiple lesions: ≥2/≤3 target lesions

	• if continuous disappearance: durable CR<br>• if measurable enhancing: preliminary PD/ pseudoresponse (1.MRI)<br><br>• steroid: off<br>• clinical: improved/stable	• if PD: preliminary PD/ pseudoresponse (1.MRI)<br><br>• steroid: stable/reduced<br>• clinical: stable/improved	• steroid: stable/improved<br>• clinical: stable	2. and 3.MRI (separated by ≥ 4 weeks): only within 12 of completion of radiotherapy:<br>• both exhibiting ≥40% increase in total volume: confirmed PD<br>• if SD or PR/CR: pseudoprogression (1.MRI)<br><br>• steroid: stable/increased<br>• clinical: deterioration	

RANO: Response Assessment in Neuro-Oncology, mRANO: modified Response Assessment in Neuro-Oncology, CR: complete response, PR: partial response, SD: stable disease, MRI: magnet resonance imaging, FLAIR: fluid attenuated inversion recovery, RT: radiotherapy.

* Threshold for volumentric change in non-enhancing disease was adopted from an approach previously described by Kickingereder et al.

Supplementary Figure 1: Scatter plots illustrating the correlation between PFS and OS for each assessment criterion.

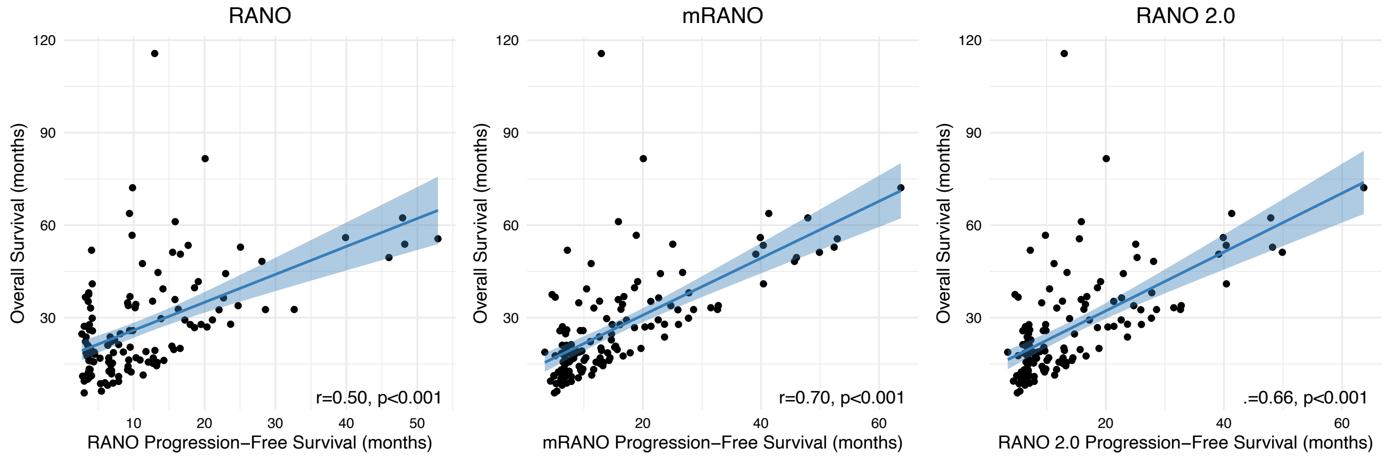

Supplementary Figure 2: Kaplan-Meier Estimates for Stable versus Progressive Disease at each Landmark.

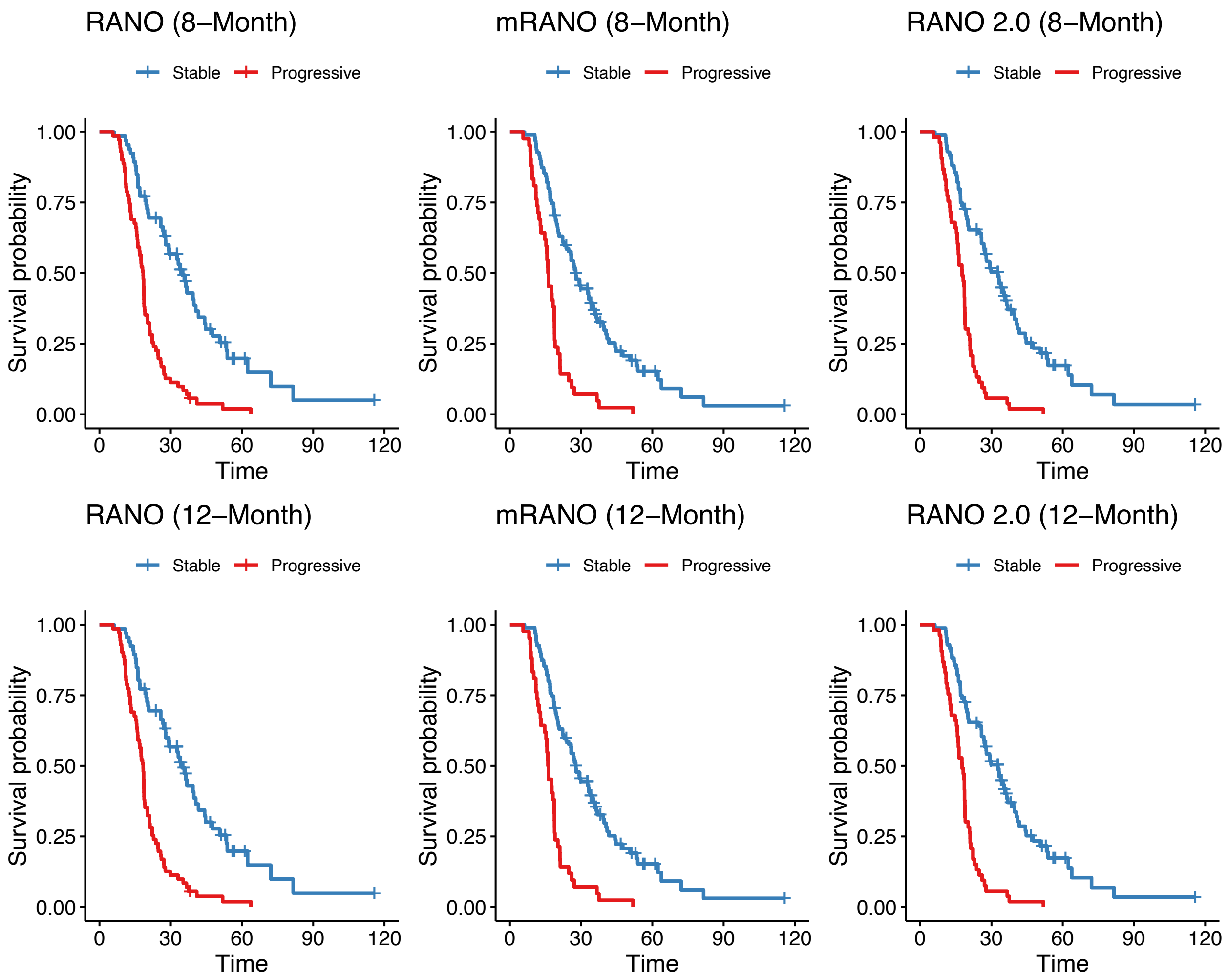

Supplement: vdag032_Supplementary_Data [file vdag032_supplementary_data.zip › 02-Mar-2026_092227_Supplementary_Revised_clear_231025.pdf]
