# Supplementary material for: Volumetric MRI-based response assessment and prognostic value in newly diagnosed glioblastoma: RANO 2.0 versus mRANO versus RANO
Source: Neurooncol Adv. 2026 Feb 12;8(1):vdag032. doi: 10.1093/noajnl/vdag032 (PMC12986764; doi:10.1093/noajnl/vdag032)
Supplement: vdag032_Supplementary_Data [file vdag032_supplementary_data.zip › Supplementary_Revised_clear_231025.docx]

Supplementary Table 1: Different Response Assessment Criteria

|  | Complete response (CR) | Partial response (PR) | Stable disease (SD) | Progressive disease (PD) |  |
| --- | --- | --- | --- | --- | --- |
| RANO | - disappearance of enhancing measurable and non-measurable - no new lesion - stable/improved non-enhancing (T2/FLAIR) - steroid: off - clinical: improved/stable | - ≥65% decrease in total volume of measurable enhancing - no progress of non-measurable - stable/improved non-enhancing (F2/FLAIR) - steroid: stable/reduced - clinical: stable/improved | - stable non-enhancing (F2/FLAIR) - stable enhancing - no new lesion - best response for patients with non-measurable disease at baseline - steroid: stable/improved - clinical: stable | - ≥40% increase in total volume of enhancing - new measurable lesion - 100% increase in total volume in non-enhancing (T2/FLAIR)* - change from non-measurable to measurable lesion - steroid: stable/increased - clinical: deterioration | - baseline MRI: after surgery - measurable disease: ≥1 cm^3^ - increase in non-measurable: >5mm or ≥25% sum of products - strong recommendation against considering PD in first 12 weeks of RT 🡪 follow-up MRI |
| mRANO | 1.MRI: Preliminary CR   - disappearance of all measurable and non-measurable disease - no new lesion   2.MRI (4-8 weeks later):   - if continuous disappearance: durable CR - if measurable enhancing: preliminary PD/ pseudoresponse (1.MRI) - steroid: off - clinical: improved/stable | 1.MRI Preliminary PR   - ≥65% decrease in total volume of measurable enhancing - no new lesion   2.MRI (4-8 weeks later):   - if SD, PR or CR: durable PR - if PD: preliminary PD/ pseudoresponse (1.MRI) - steroid: stable/reduced - clinical: stable/improved | - stable enhancing - no new lesion - best response for patients with non-measurable disease at baseline - steroid: stable/improved - clinical: stable | 1.MRI: Preliminary PD   - new measurable lesion - ≥40% increase in total volume of enhancing   2.MRI (4-8 weeks later):   - ≥40% increase in total volume: confirmed PD - if SD or PR/CR: pseudoprogression (1.MRI) - steroid: stable/increased - clinical: deterioration | - baseline MRI: after radiotherapy - no T2/FLAIR - only confirmed PD stop therapy - PD date is backdated to when first assumed |
| RANO 2.0 | 1.MRI: Preliminary CR   - disappearance of all measurable, non-measurable and nontarget disease - no new lesion   2.MRI (4-8 weeks later):   - if continuous disappearance: durable CR - if measurable enhancing: preliminary PD/ pseudoresponse (1.MRI) - steroid: off - clinical: improved/stable | 1.MRI Preliminary PR   - ≥65% decrease in total volume of measurable enhancing - no new lesion - no increase in nontarget or non-measurable lesions   2.MRI (4-8 weeks later):   - if SD, PR or CR: durable PR - if PD: preliminary PD/ pseudoresponse (1.MRI) - steroid: stable/reduced - clinical: stable/improved | - stable enhancing - no new lesion - no increase in nontarget or non-measurable lesions - best response for patients with non-measurable disease at baseline - steroid: stable/improved - clinical: stable | 1.MRI: Preliminary PD   - new measurable lesion - ≥40% increase in total volume of enhancing - definite leptomeningeal disease - clear progression of non-measurable or nontarget - failure to return to evaluation because of death or deteriorating condition   2. and 3.MRI (separated by ≥ 4 weeks): only within 12 of completion of radiotherapy:   - both exhibiting ≥40% increase in total volume: confirmed PD - if SD or PR/CR: pseudoprogression (1.MRI) - steroid: stable/increased - clinical: deterioration | - baseline MRI: 4 weeks (21-35 days) from the end of radiotherapy - if confirmation scan required, new measurable enhancing disease added to total volume. Only PD if confirmed by 2. MRI ≥4 weeks with additional ≥40% increase in volume - if multiple lesions: ≥2/≤3 target lesions |

RANO: Response Assessment in Neuro-Oncology, mRANO: modified Response Assessment in Neuro-Oncology, CR: complete response, PR: partial response, SD: stable disease, MRI: magnet resonance imaging, FLAIR: fluid attenuated inversion recovery, RT: radiotherapy.

* Threshold for volumentric change in non-enhancing disease was adopted from an approach previously described by Kickingereder et al.

Supplementary Figure 1: Scatter plots illustrating the correlation between PFS and OS for each assessment criterion.

Supplementary Figure 2: Kaplan-Meier Estimates for Stable versus Progressive Disease at each Landmark.

**
